# *Aeromonas piscicola* in Chilean *Salmon* Farming: Genomic Insights, Phenotypic Traits, Virulence and Field Immune Response

**DOI:** 10.3390/pathogens15040402

**Published:** 2026-04-08

**Authors:** Marcos Mancilla, Adriana Ojeda, Yassef Yuivar, Maritza Grandón, Sebastián Valderrama, Marcela Oyarzún, Horst Grothusen, Pablo Ibarra, Patricio Bustos

**Affiliations:** 1 Laboratorio de Diagnóstico y Biotecnología, ADL Diagnostic Chile, Sector La Vara s/n, Camino a Alerce, Casilla 160, Puerto Montt 5480000, Chile; aojeda@adldiagnostic.cl (A.O.); yyuivar@adldiagnostic.cl (Y.Y.); mgrandon@adldiagnostic.cl (M.G.); svalderrama@adldiagnostic.cl (S.V.); hgrothusen@adldiagnostic.cl (H.G.); pbustos@adldiagnostic.cl (P.B.); 2 TEKBios Fish Trial Center, Camino Pargua a Carelmapu km 8, Maullin 5580000, Chile; marcela.oyarzun@tekbios.cl (M.O.); pablo.ibarra@tekbios.cl (P.I.)

**Keywords:** *Aeromonas piscicola*, furunculosis, lipopolysaccharide, antigen, vaccine, diagnostics

## Abstract

The incidence of furunculosis in juvenile Atlantic salmon, *Salmo salar*, has increased in recent years in Chile, with isolates of *Aeromonas salmonicida* being the primary cause. However, in some cases, molecular diagnostics failed to identify the etiological agent. We previously demonstrated that a proportion of undiagnosed cases was produced by a new *A. salmonicida* strain. In those cases where the pathogen remained unidentified, we isolated colonies with an *A. salmonicida*-like appearance. Subsequent phylogenetic analysis presented in this work grouped those *A. salmonicida*-like isolates within the Aeromonas piscicola clade. Whole genome sequencing confirmed the taxonomic affiliation, giving additional insights into virulence and antibiotic resistance markers. Indeed, one of the strains showed reduced susceptibility to oxytetracycline. Virulence potential was assessed by in vivo testing in *S. salar*, which resulted in disease with pathognomonic signs of furunculosis. Although the pathogen presents common antigens with *A. salmonicida*, the current vaccine triggered only a modest IgM response against *A. piscicola* in the field. Our results support the hypothesis that the increasing incidence of furunculosis in Chile cannot solely be ascribed to the emergence of the new less-virulent *A. salmonicida* strain, but may partially result from furunculosis-like infections caused by *A. piscicola* strains which exhibit a comparable virulence level.

## 1. Introduction

Members of the bacterial genus *Aeromonas* are natural inhabitants of aquatic environments, including freshwater, estuarine, and marine water bodies. Several species are pathogenic for fish, causing ulcerative, hemorrhagic and even septicemic diseases with relevant economic losses [1]. Among those diseases, furunculosis is commonly associated with skin lesions which rapidly progress to a septicemic infection with high mortality, typically caused by members of the *Aeromonas salmonicida* clade [2]. Classical furunculosis is produced by brown-pigmented, psychrophilic, Gram-negative bacteria, grouped into the subspecies *A. salmonicida* subsp. *salmonicida* which mainly affects wild and cultured salmonids. By contrast, non-pigmented atypical subspecies are able to infect a broader number of hosts including carp (*Cyprinus carpio*), goldfish (*Carassius auratus*), flounder (*Platichthys flesus*), Siberian sturgeons (*Acipenser baerii*), and captive snakehead fish (*Channa argus*) [2,3,4,5], with the exception of *A. salmonicida* subsp. *pectinolytica*, a mesophilic subspecies that was only isolated from environmental samples [6].

*Aeromonas piscicola* is closely related to *A. salmonicida*. This bacterium has first been isolated from cultured salmonids in Spain that exhibited signs of furunculosis-like disease. Phenotypically, *A. piscicola* comprises mesophilic, motile microorganisms that do not produce brown pigment, and which can be distinguished from mesophilic *A. salmonicida* by its inability to ferment lactose and L-arabinose. Conventional 16S rRNA sequencing, however, cannot differentiate between them [7]. More recently and along with other pathogenic members of the Aeromonadaceae family, *A. piscicola* has been associated with Red Skin Disease, a condition affecting wild salmonid populations in Europe [8]. In the context of increasingly challenging environmental conditions driven by climate change, it is important to draw attention to mesophilic bacteria, particularly pathogens affecting fish species reared in freshwater, which is especially susceptible to warming.

Chile, as the world’s second-largest salmonid producer, faces several sanitary challenges in both stages of the production cycle. In freshwater, the incidence of furunculosis in Atlantic salmon (*Salmo salar*), the most relevant reared salmonid species in the Chilean industry, has increased in recent years, from almost marginal to 7.8% of the total amount of secondary mortality (i.e., caused by infectious diseases). Still, a decline was noted in the last report [9]. Atypical *A. salmonicida* has long been recognized as responsible for furunculosis [10], but in the face of increasing incidence, some authors have suggested its re-emergence [11]. The discovery of an *A. salmonicida* strain associated with mild disease with limited mortality, which may turn more deadly when stress and suboptimal rearing conditions occur, added a new layer of complexity to the epidemiological scenario [12].

Using our improved PCR scheme based on *vapA-fstA* genes for typing *A. salmonicida* isolates from our strain collection, we observed that some *A. salmonicida*-like isolates did not amplify in the molecular assay. In the present work, we unveiled the identity and phenotypic characteristics of those isolates by combining sequencing and microbiological approaches. Our results strongly support the identification of *A. piscicola* as the causative agent of some recent furunculosis-like outbreaks, which is herein reported for the first time in Chile. We further characterized its virulence potential in Atlantic salmon and present evidence on the scarce serological response elicited by the current furunculosis vaccine in comparison with the immune response directed against *A. salmonicida*.

## 2. Materials and Methods

### 2.1. Biological Material and Culture

Tissue samples from suspected cases of furunculosis were collected during diagnostic routine at ADL Diagnostic’s laboratory in Puerto Montt, Chile. Samples, maintained at −20 °C in ethanol, were subjected to a DNA purification protocol (E.Z.N.A. tissue DNA purification kit, Omega Bio-tek, Norcross, GA, USA). Bacterial strains stored at −80 °C in the ADL strain collection were recovered and cultured on trypticase soy agar (TSA; BD) plates at 18 °C for 48 h. Some isolates were grown in trypticase soy broth (TSB; BD). For molecular and sequencing analysis, a single colony of each isolate was processed for DNA purification using a commercial kit (GeneJET genomic DNA purification kit, Thermo Scientific, Waltham, MA, USA). Metadata of bacterial strains are shown in Table 1.

### 2.2. Bacterial Growth Kinetics, Counts and Antibiotic Susceptibility Testing

Bacterial isolates were grown on TSA plates at 18 °C for 48 h. Then, a loopful of each isolate was transferred to sterile saline to prepare bacterial suspensions, which were then adjusted to OD_600_ = 1.00. Next, 96-well microplates were inoculated with 100 µL of these preparations diluted in TSB at 1:100. Bacterial growth kinetics of three *A. piscicola* and two *A. salmonicida* isolates were evaluated in an automatic spectrophotometer EPOCH 2 (BioTek, Winooski, VT, USA) at incubation temperatures of 10, 23, and 37 °C. Reads were taken hourly for 48 h. To assess the effect of salinity on growth, TSB was supplemented with 0, 0.5, and 2% of NaCl. For bacterial counts, suspensions were 10-fold serially diluted in saline, plated in triplicate onto TSA, maintained at 18 °C for 48 h, and counted.

For antibiotic susceptibility assays, a saline suspension of bacteria freshly cultured on TSA was prepared and likewise adjusted to OD_600_ = 1.00. Minimal inhibitory concentration (MIC) testing was performed using a 2-fold serial microdilution method in Müller-Hinton (M-H) broth, with concentrations ranging from 0.03 to 64 µg × mL^−1^. Antimicrobial agents florfenicol and oxytetracycline were purchased from Sigma-Aldrich Co., (St. Louis, MO, USA) and were prepared following the CLSI recommendations for broth dilution susceptibility testing of bacteria isolated from aquatic animals, guidelines VET04-A2 and VET03/VET04-S2 [13,14]. Standard stock solutions were prepared by dissolving 10 mg of each antibiotic in 500 µL of 95% ethanol and 1 M NaOH for florfenicol or 100% methanol for oxytetracycline, and the final volume was adjusted to 10 mL with sterilized Milli-Q water. Stock solutions were stored at −80 °C. Challenge trays included positive control wells containing bacterial suspensions in M-H broth without antibiotic, and negative controls consisting of uninoculated broth. Each test was carried out in triplicate, considering incubation for 48 h at 18 °C. The MIC was defined as the lowest concentration of the antibiotic that prevented bacterial growth.

### 2.3. PCR Assays and Whole Genome Sequencing

Specific TaqMan qPCR assays for *vapA-fstA* genes were performed for the identification of *A. salmonicida* according to our previous work [12]. The same validated AgPath-ID One-Step RT-PCR chemistry used in our routine diagnostic workflow was maintained for these assays, although DNA was the nucleic acid template in this study. PCR conditions were as follows: For every primer/probe set, 300 nM of primer and 200 nM of probe were combined and mixed with 3 µL of nucleic acid template, enzyme and master mix using AgPath-ID™ One-Step RT-PCR and nuclease-free water as per the manufacturer’s instructions (Applied Biosystems™, Carlsbad, CA, USA). Furthermore, we considered 10 min at 45 °C for reverse transcription and 10 min at 95 °C for reverse transcriptase inactivation, followed by 45 cycles of 5 s at 95 °C and 30 s at 60 °C for annealing-extension. Reactions were run in a Quant Studio 3 PCR machine (Applied Biosystems™). A similar amplification protocol was followed for examining tissue samples derived from the bioassay, employing a primer set for *Aeromonas* spp. described elsewhere [15]. Sequences of primers and probe set are listed in Table S1.

Whole genome sequencing was conducted at Codebreaker Bioscience (Santiago, Chile). Libraries were created using the standard protocol of Nextera Flex (Illumina™, San Diego, CA, USA). All standard reagents from the Nextera DNA Flex library prep kit were used to prepare each standard Flex library, in accordance with the manufacturer’s instructions. A Qubit high-sensitivity (HS) dsDNA kit (Thermo Fisher Scientific, Waltham, MA, USA) and the S2 Cartridge from BIOtic (Pinneberg, Germany) with the Qsep1 Machine were used to assess the concentration of eluted libraries and the library size. For sequencing, a 600-cycle kit configured for 2 × 300 bp paired-end reads was used on an Illumina™ Nextseq1000™ platform.

### 2.4. Bioinformatic Analysis

A custom Nextflow pipeline was applied to analyze raw FASTQ files https://github.com/gene2dis/mgap (accessed on 24 February 2026). That pipeline included quality control checks and cleaning of the raw reads with FastP (v.0.23.2) [16], assembly with Spades (v.3.15.5) [17] and contig annotation with Bakta (v.1.7.0) [18]. Finally, we employed BLASTN v2.15.0 to screen public databases for homologous sequences of contigs of interest [19]. Furthermore, the clinker tool described by Gilchrist and Chooi [20] allowed us to visualize groups of homologous biosynthesis gene clusters. Average nucleotide identities (ANI) were calculated with the ANI calculator tool hosted at https://www.ezbiocloud.net/ (accessed on 25 February 2026) [21]. Phylogenomic analysis was conducted using the Type Strain Genome Server [22] to compare A2, S2, and S3 strains with sequences of reference type strains from the DSMZ database including *A. salmonicida* CIP 103209, *A. salmonicida* subsp. *oncorhynchi* A-9, *A. salmonicida* subsp. *masoucida* NBRC 13784, *A. salmonicida* subsp. *achromogenes* JCM 7875, *A. salmonicida* subsp. *pectinolytica* DSM 12609 (two assemblies), *A. hydrophila* ATCC 7966, *A*. *hydrophila* subsp. *ranae* CIP 107985, *A. piscicola* LMG 24783, *A. sobria* CECT 4245, *A. molluscorum* CECT 5864, *A. bestiarum* CECT 4247, *A. encheleia* CECT 4342, and *A. popoffii* CIP 105493.

The functional genome characterization of A1, A2, A4, S2, and S3 was conducted using customized Python v.3.12.13-based bioinformatics pipelines run in Google Colab (https://colab.google/, accessed on 24 February 2026) to predict virulence factors (VF) and antibiotic resistance genes. Virulence-associated genes were inferred by screening the genomes against the Virulence Factor Database (VFDB) v5 [23]. This database contains curated coding sequences (CDS) of genes categorized into toxins, secretion systems, adhesion factors, and immune evasion mechanisms. Reciprocal Best Hit (RBH) analysis was performed to validate VF identification. Briefly, the VFDB protein dataset was indexed to create a DIAMOND reference database. Each *.faa file was subjected to a DIAMOND BLASTP v.2.0.14 search against the prepared VFDB. This step identified initial best hits from the query genome to VFDB entries. Hits from the forward search were filtered using stringent criteria: minimum 60% protein sequence identity (MIN_PIDENT), minimum 90% query and subject coverage (MIN_COV), maximum E-value of 1 × 10^−50^ (MAX_EVALUE), and a minimum alignment length of 100 amino acids (MIN_ALN). A subset of the VFDB was dynamically generated, containing only the VF entries that passed the strict filtering from the forward search. Concurrently, a DIAMOND database was created from the original query genome’s proteome. A reverse DIAMOND BLASTP search was then performed, querying the VFDB subset against the query genome database. The results from the strictly filtered forward and reverse searches were merged. Only hits that were RBH (i.e., protein A from genome is the best hit for VF B, and VF B is the best hit for protein A) were retained as putative VF orthologs. The identified RBH pairs were annotated with their corresponding VFDB header, gene symbol (extracted from the header when available), VFDB Group ID, and a robust VF_key for consistent identification across genomes. A sequence with a known number of VF was used as calibrator [24].

Protein FASTA files from bacterial genomes were then processed to identify antimicrobial resistance (AMR) genes using the AMRFinderPlus tool v.4.2.7 [25], with results compiled into a Pandas data frame. For comparative validation, AMRFinderPlus outputs were cross-referenced with the RGI v.6.0.7 of Comprehensive Antibiotic Resistance Database (CARD) prediction dataset [26].

### 2.5. Purification and Analysis of Bacterial Membrane Antigens

Outer membrane protein (OMP) fractions were obtained following a modified, Sarkosyl-based extraction strategy [27]. Briefly, isolates grown on TSA were suspended in saline and subsequently centrifuged at 10,000× *g* for 10 min. Pellets were resuspended in lysis buffer containing 300 mM NaCl, 10 mM HEPES, 2 mM PMSF, and 8 M urea, before sonication on ice for 1 min at 70% potency (300 V/T, Biologics Inc., Cary, NC, USA). Crude extracts thus obtained were centrifuged at 10,000× *g* for 10 min at 4 °C. They were resuspended in 1 mL of 0.2% Sarkosyl and incubated overnight with agitation at room temperature (RT). These samples were centrifuged at 10,000× *g* for 1 h at 4 °C. Pellets were then washed twice with 10 mM Tris-HCl (pH 7.4) and recovered by centrifugation at 10,000× *g* for 10 min at 4 °C. Lastly, OMP fractions were resuspended in buffer containing 10 mM Tris-HCl (pH 7.4) and 8 M urea. Proteins were stored at −20 °C until use.

We adapted the method described by Yi and Hackett [28] in order to isolate and purify lipopolysaccharide (LPS). Bacteria grown on TSA were suspended in 300 µL of Trizol reagent and incubated for 15 min at RT. Next, 100 µL of chloroform was added. Samples were mixed thoroughly and incubated for another 10 min, maintaining RT. Centrifugation of the water phase at 12,000× *g* for 10 min was followed by treatment with 500 µL of 0.375 M MgCl_2_ in cold ethanol (−20 °C). Pellets were recovered by a final centrifugation step at 12,000× *g* for 15 min at 4 °C. Discontinuous SDS-PAGE electrophoresis was performed to separate membrane antigens. We used 5% and 15% polyacrylamide stacking/solving gels which were subsequently stained with Coomassie blue dye to assess proteins; silver stains were used for LPS analysis, following a protocol described elsewhere [29]. We ran semi-dry Western blots (Bio-Rad, Dreieich, Germany), revealing signals with a polyclonal antibody obtained from rabbit serum after immunization with a *A. salmonicida* VapA+ isolate and the corresponding HRP-conjugated secondary antibody.

### 2.6. Virulence In Vivo Testing

*Salmo salar* with a mean weight of 28 g were intraperitoneally challenged with either one of three infectious doses of representative *A. piscicola* field isolates (~10^6^, ~10^7^, or ~10^8^ cfu/fish of A2 or S2, respectively; for exact counts, see Table S2). Bacterial suspensions were freshly prepared as described in Section 2.2, including a wash step with saline before final resuspension. The assay was conducted in a single tank containing 500 L of filtered, UV-treated freshwater. We worked with a total of 140 fish: 20 healthy control fish (14.3%) which received an intraperitoneal injection of 0.1 mL saline and 120 fish (85.7%) to be injected with bacterial inoculum. All experimentally challenged and control fish were maintained in that same tank. The 120 fish to be challenged were divided into six subgroups and marked accordingly by Visible Implant Elastomer (VIE tagging), and each subgroup was treated with *A. piscicola* A2 or S2 strains at a determined infectious dose. Fish biomass density was kept below 30 kg/m^3^. The experiment was carried out at 10.7 ± 0.1 °C and 4.0 ppt salinity, and it was ended 30 days post-inoculation (dpi). Mortality was registered daily, and pooled samples of anterior kidney, liver, and spleen were collected from each deceased fish. These samples were stored in tubes with RNAlater (Life Technologies, Carlsbad, CA, USA) and kept at −80 °C until further use. At 30 dpi, all survivor fish, including controls, were sacrificed to obtain internal organ samples for PCR analysis. Pathogen re-isolation was not performed during the challenge; infection status was assessed by qPCR on pooled internal-organ samples.

### 2.7. Serum Immune Response

In preparation for a field study intended to shed light on the immune response to *A. piscicola*, we collected serum samples from *S. salar* on seven fish farms in Southern Chile where a commercial pentavalent vaccine against atypical furunculosis, Infectious Pancreatic Necrosis (IPN), Infectious Salmon Anemia (ISA), Salmonid Rickettsial Syndrome (SRS), and vibriosis caused by *Vibrio ordalii* was to be applied. Serum samples were taken from five fish per farm at different time points immediately before and after vaccination: First, unvaccinated *S. salar* pre-smolts were euthanized applying an overdose of benzocaine. These fish were bled to obtain serum samples, which were subsequently centrifuged at 1000 rpm for 3 min. Further fish were sampled during the freshwater stage at ~300 and ~600 thermal units (TU) time points post vaccination. Immune responses were further monitored post sea transfer with additional samples being collected at ~900 and ~1200 TU post vaccination. All serum samples were stored at −80 °C until further analysis.

To assess immune responses, 96-well microplates were coated with 5 µg of bacterial protein purified from the representative isolates *A. piscicola* S2 (*vapA−*) or *A. salmonicida* A4 (*vapA+*). Enzyme-linked immunosorbent assays (ELISA) were performed in these pretreated microplates, using 5% skim milk as the blocking agent and PBS containing 0.05% Tween (PBS-T) as the washing buffer. Fish serum was diluted 1:100 in skim milk and added to the wells. Loaded microplates were incubated for 1 h at RT, washed with PBS-T, incubated with a monoclonal HRP-conjugated antibody anti-salmon IgM, and washed again. Signals were developed using the chromogenic substrate 3,3′,5,5′-tetramethylbenzidine and measured as absorbance at 450 nm with a spectrophotometer.

### 2.8. Statistical Analysis and Plots

Plots were generated using the ggplot2 package on R (version 4.0.5). The Kaplan–Meier method was used to analyze cumulative mortality percentages in the in vivo assay, and the differences were evaluated using log-rank. Statistical tests were also performed with R.

## 3. Results

### 3.1. Bacterial Strains, PCR Typing and Phenotypic Profiling

ADL’s strain collection comprises *A. salmonicida-*like isolates that have been collected during freshwater furunculosis outbreaks since 2022 (Table 1). Beyond that, we included two *A. salmonicida* isolates depicting distinctive A-layer phenotypes for comparative purposes. All bacterial specimens were obtained from diseased *S. salar* or rainbow trout, *Oncorhynchus mykiss*. None of these isolates amplified in qPCR assays using *vapA* and *fstA* as gene markers specific for *A. salmonicida* species.

**Figure 1:**
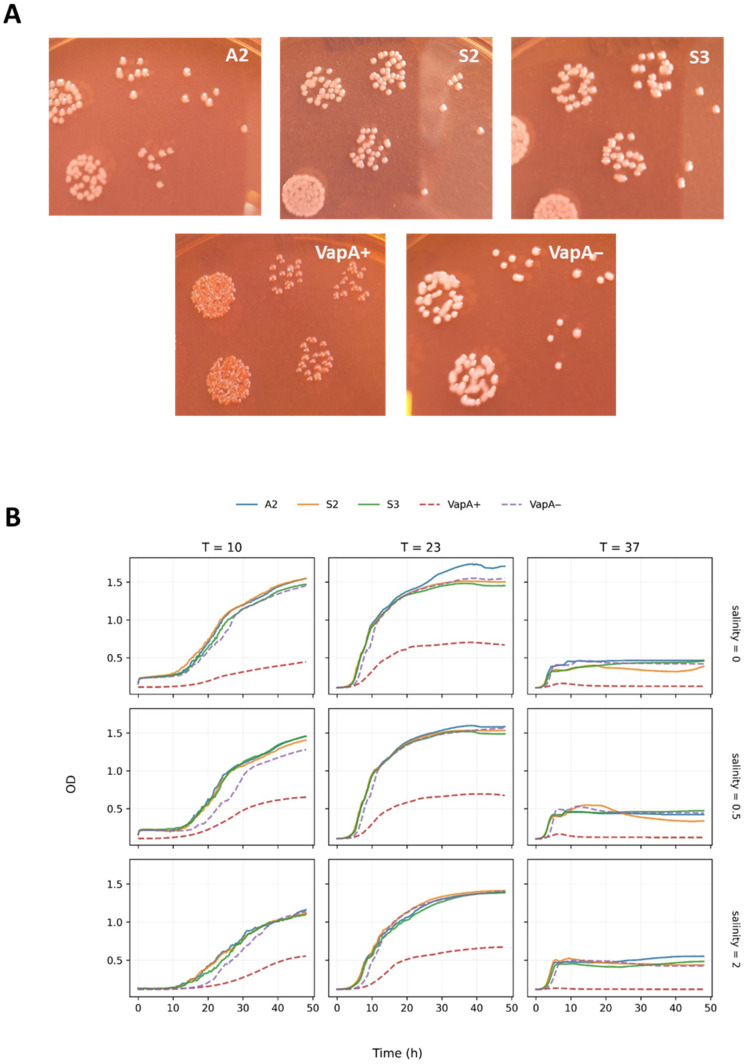
Phenotypic characterization of *A. salmonicida-*like isolates. (**A**) Bacterial colonies on TSA plus Congo red. (**B**) Growth kinetics on TSB at different temperatures (°C) and salinities (added NaCl [g/dL]). VapA+ and VapA− correspond to the A4 and A1 isolates listed in Table 1, respectively.

The A-layer phenotype was examined using a classical culture method, exploiting Congo red retention by bacteria forming this membrane structure [30]. As illustrated in Figure 1A, neither the reference *A. salmonicida vapA*-absent isolate A1 nor *A. salmonicida-*like isolates (A2, S2 and S3) are able to adsorb the dye and thus remain gray. Because Congo red retention depends on A-layer protein expression, our observations suggest that the colonies lack this molecule. This finding is consistent with the *vapA* PCR result. For further phenotypic characterization, we assessed these colonies’ growth at different temperatures and salinities (Figure 1B). The corresponding growth curves reveal optimal growth for all isolates at mesophilic temperatures (23 °C), maintaining the ability to adapt to psychrophilic conditions (10 °C). Growth at 37 °C, albeit suboptimal, could be confirmed for the *A. salmonicida vapA-*absent genotype and for all *A. salmonicida-*like isolates. Notably, isolate *A. salmonicida* A4, a representative of the more “classical” *vapA+* version of *A. salmonicida*, exhibited poorer growth under all tested conditions. Salinity had little effect on growth across the tested range (0–2%).

Regarding antibiotic susceptibility to the main drugs used in Chilean salmon farming to control bacterial infections, *A. salmonicida-*like isolates showed MIC values ranging from 0.25 to 1.0 µg/mL for both florfenicol and oxytetracycline, with the exception of the A2 isolate, which exhibited a higher MIC value for oxytetracycline (>64 µg/mL; Table 2).

### 3.2. Phylogenetic and Bioinformatic Analysis

**Figure 2:**
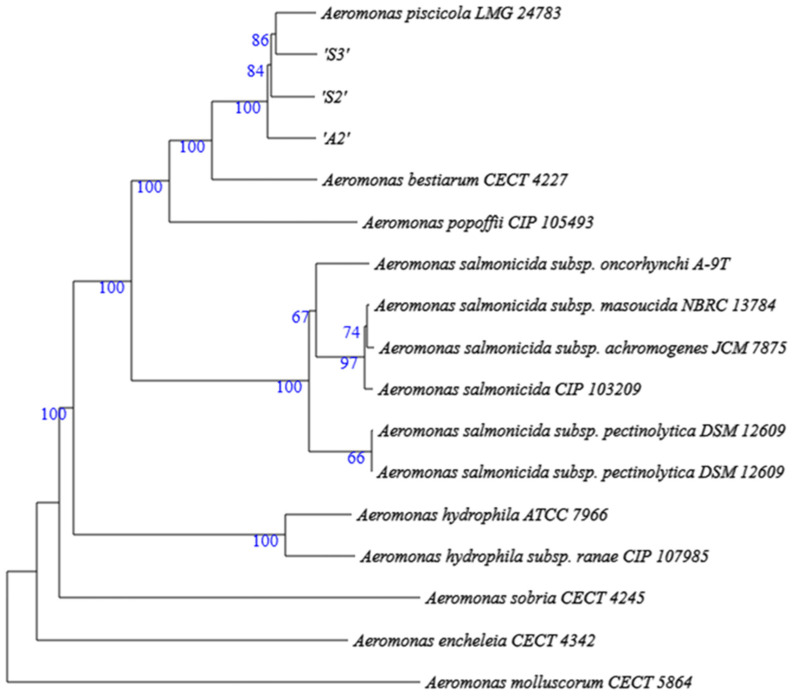
Phylogenetic tree of Chilean *A. piscicola* isolates based on Genome BLAST Distance Phylogeny using TYGS. Bootstrap values > 60% (100 replicates) are shown at nodes.

The sequences of all draft genomes were deposited in EMBL, BioProject PRJEB105947. Table 3 shows the corresponding accession numbers. Genome-based phylogeny placed the three *A. salmonicida*-like isolates A2, S2, and S3 in the same clade of genomes, namely that of *A. piscicola* type strain LGM 24783, with an average branch support of 86% and clustering very close to *A. bestiarum* and *A. popoffii* (Figure 2). However, comparative analyses using different methodological approaches revealed remarkable differences in their respective genome contents, such as the number of CDS predicted to be VF (Table 4 and Table S3). In this regard, all *A. piscicola* genomes were shown to carry about 220 genes. This represents an approximately one-third increase in the total VF repertoire compared with 151 VF of the reference type strain *A. salmonida* subsp. *oncorhynchi* A-9 [24], positioning Chilean *A. salmonicida* genovariant genomes in a second place with total counts of 164 and 186, respectively. The identified VF categories comprise factors involved in host adherence, biofilm formation, surface antigens, exoenzymes, iron uptake, motility, toxins, and secretion systems. Notable, within the latter category, we want to highlight that Type 3 Secretion System (T3SS) genes were found in the genomes of all Chilean *A. piscicola* isolates and *A. salmonicida vapA+* strain A4, while they were absent from the genome of the reference type strain *A. salmonida* subsp. *oncorhynchi* A-9 and *A. salmonicida vapA−* strain A1 (Table 5).

**Figure 3:**
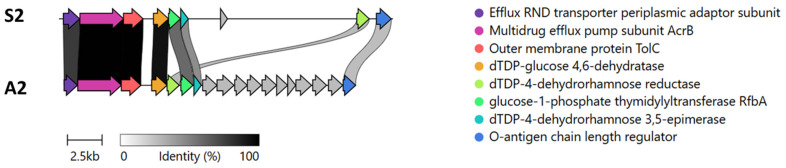
O-antigen biosynthesis gene cluster in the *A. piscicola* A2 genome. Predicted functions for each CDS are color-coded and specified in the legend. In gray, those CDS encoding proteins involved in O-antigen biosynthesis. Predicted functions for CDS in gray are found in Table S4.

Regarding genes related to bacterial surface composition, we identified an indel of ~10 kb in the genome of the *A. piscicola* strain A2 (Figure 3). This locus encodes genes that appear to constitute a novel O-antigen biosynthesis cluster and was not detected in any other microorganism listed in the GenBank database according to BLAST searches, with the exception of *Aeromonas veronii* AGM2 (Table S4). On the other hand, the prediction of antibiotic resistance genes revealed high similarity to a unique AMR gene, which codes for tetracycline efflux major facilitator superfamily (MFS) transporter *tet(E)*. This gene has been related to tetracycline resistance in *Escherichia coli* [31].

### 3.3. Membrane Antigen Profiling

**Figure 4:**
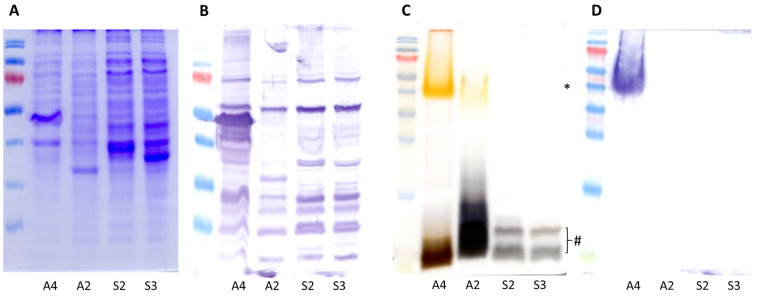
Outer membrane component profiling of *A. piscicola* isolates. (**A**) OMP profiles. (**B**) OMP Western blot using serum from a rabbit hyperimmunized against *A. salmonicida vapA+*. (**C**) LPS patterns in silver-stained SDS-PAGE. Low- and high-molecular-weight LPS bands are indicated with # and *, respectively. (**D**) LPS Western blot revealed with the same antibody used in B. Lane IDs refer to bacterial isolates as described in Table 1.

OMP profiles corroborated specific patterns for all tested isolates, including *A. salmonicida vapA+* A4 as a reference (Figure 4A). However, further analysis concerning the protein fraction reactive to serum from rabbits immunized with *A. salmonicida vapA+* revealed common bands among all isolates, with two clearly distinct patterns emerging for *A. piscicola* isolates, namely A2 versus S2 and S3 (Figure 4B). Similarly, the LPS profiles of isolates allowed their classification into two groups: *A. salmonicida vapA*+ and *A. piscicola* A2 presented a dual-band pattern which is structurally concordant with the presence of lipid A-core (low molecular size) plus O-antigen polysaccharide (high molecular size). By contrast, S2 and S3 isolates exhibited identical bands of low molecular size profile. The lowest band presumably corresponds to lipid A-core oligosaccharide, while the slightly heavier band likely represents the lipid A-core plus single O unit (Figure 4C). A single high-molecular-weight band was detected in LPS Western blots exclusively for the *A. salmonicida vapA*+ A4 reference strain (Figure 4D). This finding is in accordance with the homologous nature of the polyclonal antibody used to reveal the signal.

### 3.4. In Vivo Virulence

**Figure 5:**
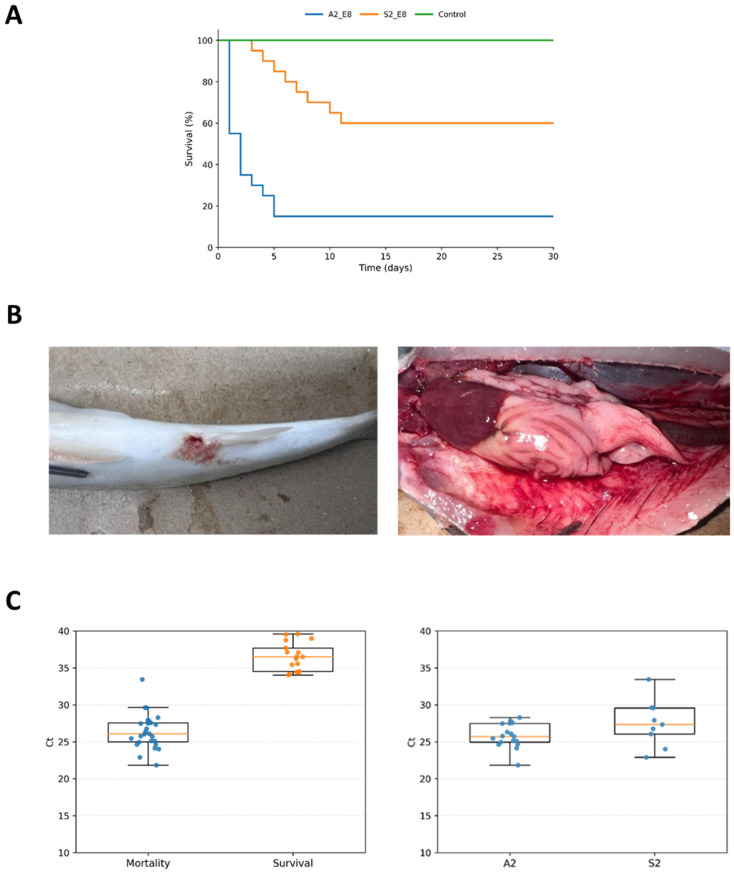
In vivo virulence of *A. piscicola* isolates in *S. salar*. (**A**) Survival analysis of fish inoculated with A2 or S2 isolates using ~10^8^ cfu/fish (A2_E8, S2_E8), or saline (Control). Of note, survival curves for fish challenged with ~10^6^ or ~10^7^ cfu/fish overlap with those of negative controls. (**B**) Gross pathological findings in Atlantic salmon: Left panel, focal ulcerative-hemorrhagic skin lesion on the flank. Right panel, open coelomic cavity showing severe visceral congestion and hemorrhage, with hyperemic intestine and congested liver. (**C**) Relative bacterial loads in internal organs measured by PCR targeting *Aeromonas* spp. Box and whisker plots, left panel: Mortality and Survival groups of fish tested, including controls. Right panel: Ct values registered for strain-specific Mortality subgroups for both *A. piscicola* isolates tested.

Although both *A. piscicola* isolates, namely A2 and S2, were able to kill fish, the intraperitoneal infection model revealed a marked difference in virulence. The *A. piscicola* isolate A2 killed fish from day one, while fish inoculated with the S2 strain did not show mortality until three dpi. This is consistent with the observation that a mortality rate of more than 80% was reached as fast as four dpi in *S. salar* inoculated with the highest dose of A2 (~10^8^ cfu/fish, Figure 5A). By contrast, the mortality rate of fish challenged with the corresponding dose of S2 did not exceed 45%, and that value was not reached until 11 dpi. Concerning the disease phenotype in dead fish, further examination showed focal cutaneous ulcerative lesions, marked visceral congestion, and inflammation consistent with acute systemic vascular injury and septicemic disease (Figure 5B). No mortality was registered for lower doses, regardless of the isolate. Neither A2 nor S2 were able to infect cohabitant control fish during the assay, indicating a limited dissemination capacity. Relative bacterial loads were determined by PCR using pooled kidney, liver, and spleen samples from each individual fish, including survivors and mortalities. Results show that both isolates were able to infect internal organs, most likely inducing death due to septicemic disease (Figure 5C).

### 3.5. Immune Response of Vaccinated Fish

**Figure 6:**
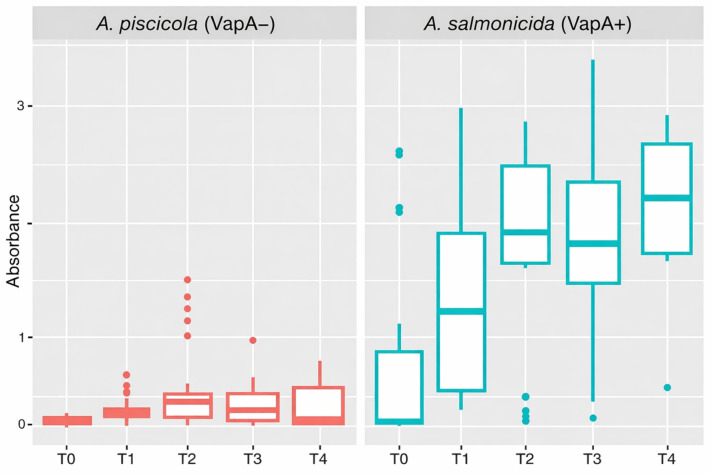
Serum immune response against *A. piscicola* (ELISA). Fish derived from seven farms were sampled at 0 (T0), ~300 (T1), ~600 (T2), ~900 (T3) and ~1200 (T4) TU after vaccination. Relative IgM levels were examined in independent microplates coated with purified protein of *A. piscicola* (VapA−) or *A. salmonicida* (VapA+).

*A. piscicola* S2-reactive and *A. salmonicida* A4-reactive serum IgM levels were quantified by ELISA using microplates coated with the respective bacterial proteins. Combined analysis of all tested fish showed that vaccinated individuals developed progressively increasing antibody levels against *A. salmonicida vapA+* in freshwater (up to ~600 TU), consistent with the homologous antigen present in the vaccine formulation (atypical *A. salmonicida*). Indeed, the level of these antibodies remained high even post sea transfer (Figure 6, right panel). Conversely, and consistent with its heterologous nature, anti-*A. piscicola* IgM levels remained lower in the same fish, even at later stages (Figure 6, left panel).

## 4. Discussion

This study provides evidence that *A. piscicola* is associated with recent furunculosis-like outbreaks in Chile and that isolates can cause mortality in Atlantic salmon under experimental conditions, a fact that adds complexity to the epidemiological scenario of the disease. The detection of *A. piscicola* in Chile raises concerns regarding its potential role as a pathogen in aquatic systems. Recent reports from other countries have associated *A. piscicola* with infections in *S. salar*, causing symptoms such as hemorrhagic septicemia and skin ulcerations [8]. The bacterium’s adaptation to the prevailing environmental conditions in Chile highlights its resilience and potential for dissemination. In fact, our evidence suggests that *A. piscicola* isolates, as well as *A. salmonicida vapA*-absent variants, are more versatile than the long-known, highly virulent *A. salmonicida vapA+* strain: They are well able to replicate in a wide range of temperature and salinities. Our findings underscore the need for surveillance programs to monitor the presence of *A. piscicola* in aquaculture facilities and natural water bodies. Current diagnostic strategies are only focused on the detection of *A. salmonicida*, based on the assumption that only one species (and its variants) is responsible for furunculosis in reared salmon in Chile. However, this work expands our knowledge, highlights the virulence potential of *A. piscicola* strains, and thus points out the need to update diagnostic procedures. Ongoing research is addressing this issue; we are developing specific molecular assays to better distinguish *A. salmonicida* variants as well as non-*salmonicida* isolates such as *A. piscicola*.

A striking finding of this study is the extensive repertoire of VF identified in *A. piscicola* genomes. With approximately 220 predicted virulence genes, these isolates possess a pathogenic arsenal roughly one-third larger than the reference *A. salmonicida* subsp. *oncorhynchi* A-9. Notably, T3SS genes have been identified in all *A. piscicola* isolates, whereas they are characteristically absent from the *A. salmonicida* subsp. *onchorynchi* A-9 reference genome. At the same time, this observation should not be generalized to all representatives of the *A. salmonicida* species, because T3SS determinants have been reported in other *A. salmonicida* genomes [32], consistent with our observation in the genome of Chilean *A. salmonicida vapA+* strain A4. Therefore, the present comparison should be interpreted as reference-genome-based rather than species-wide, and a more comprehensive comparison against multiple typical and atypical *A. salmonicida* genomes will be required to determine whether the differences observed here are species-level or lineage-specific. Virulence is a complex biological phenomenon that is not determined solely by the number of VF, and hence the total number of VF identified in *A. piscicola* genomes cannot support the notion that *A. piscicola* isolates described here are more virulent than their *A. salmonicida* counterparts.

Unlike classical *A. salmonicida*, the *A. piscicola* isolates described herein were mesophilic, vapA-negative, and antigenically distinct in their OMP/LPS profiles. Furthermore, structural and genomic differences in the outer membrane composition provide a plausible explanation for the observed variations in virulence among the *A. piscicola* strains themselves. The highly virulent A2 isolate (causing >80% mortality) was shown to possess a complete LPS structure featuring both the lipid A-core and a high-molecular-weight O-antigen. Conversely, the less virulent S2 isolate exhibited a truncated LPS profile lacking the polymeric O-antigen fraction. This phenotypic divergence is strongly supported by our genomic analysis, which revealed a unique ~10 kb O-antigen biosynthesis gene cluster in the A2 genome that is absent in the *A. piscicola* type strain LMG 24783. The presence of this novel O-antigen likely enhances serum resistance and host colonization, making A2 significantly more lethal than the S2 variant. This feature may also explain the early mortality observed in fish inoculated with what appears to be a bacterium carrying a more biologically active LPS, although the precise contribution of this locus to virulence require functional validation.

These two strains also differ with regard to antimicrobial susceptibility: While the majority of the mesophilic *A. piscicola* isolates were confirmed to be susceptible to the primary antibiotics used in Chilean salmon farming, the A2 strain demonstrated pronounced resistance to oxytetracycline (MIC > 64 µg/mL). Our bioinformatic analysis successfully linked this phenotype to the presence of the *tet(E)* MFS efflux pump gene. The circulation of such resistance markers within a virulent, environmentally versatile strain poses a severe risk to standard therapeutic interventions in freshwater aquaculture, emphasizing the critical need for continuous AMR monitoring.

From an immunological and preventative standpoint, our results reveal a significant vulnerability in current prophylactic strategies. The ELISA data clearly demonstrate that the commercial pentavalent vaccine, which relies on homologous *A. salmonicida* antigens (such as the A-layer protein VapA), elicits a robust IgM response against classical *A. salmonicida* but fails to trigger a comparable cross-reactive response against *A. piscicola* in the field. This immune evasion is almost certainly due to the heterologous nature of the outer membrane of *A. piscicola*, which lacks the VapA protein and, in the case of the A2 isolate, expresses a highly divergent, unrecognized O-antigen, as confirmed by our Western blot analysis (Figure 4D). Consequently, vaccinated Atlantic salmon remain immunologically naïve to *A. piscicola*, facilitating the outbreaks observed in recent years.

In conclusion, the emergence of virulent, mesophilic *A. piscicola* strains capable of circumventing current commercial vaccines represents a paradigm shift in the management of furunculosis-like diseases in Chile. Future efforts must prioritize the development of multivalent or autogenous vaccines that incorporate *A. piscicola*-specific antigens, alongside the implementation of multiplexed diagnostic tools capable of accurately differentiating between these closely related, yet functionally distinct, *Aeromonas* species.

## Figures and Tables

**Table 1:** Metadata related to bacterial strains used in this work, all of which were recovered from freshwater. PCR results for *vapA* and *fstA* markers are shown as positive (p) or negative (n). Ss, *Salmo salar*; Om, *Oncorhynchus mykiss*.

ID	Routine Code	Year Isolation	Stage	Host	Region	Tissue	*vapA*	*fstA*	Reference
A1	PM-118565	2022	Fry	Ss	X	Skin lesion	n	p	[12]
A2	PM-117383	2022	Fry	Ss	X	Skin lesion	n	n	This work
A4	PM-118328	2022	Smolt	Ss	X	Kidney	p	p	[12]
S2	PM-123729	2022	Fry	Om	VIII	Skin lesion	n	n	This work
S3	PM-124921	2023	Fry	Ss	IX	Gill	n	n	This work

**Table 2:** Strain-specific minimal inhibitory concentrations (µg/mL) of florfenicol (FFC) and oxytetracycline (OTC).

Strain ID	FFC	OTC
A2	0.50	>64.0
S2	0.50	0.25
S3	1.00	0.50

**Table 3:** BioSample accessions for draft genomes (Bioproject PRJEB105947).

Accession	Sample Name	Organism	Tax ID
ERS28383287	A2	*Aeromonas piscicola*	600645
ERS28383288	S2	*Aeromonas piscicola*	600645
ERS28383289	S3	*Aeromonas piscicola*	600645

**Table 4:** Virulence factors found in the genome of *A. piscicola* and selected *A. salmonicida* strains, and reference type strains LMG 24783 and A-9 (category and CDS counts).

Genome	Adhesion (Pili, Fimbriae)	Biofilm, Quorum Sensing	Capsule, LPS, Surface	Exoenzymes (Tissue Degradation)	Iron Uptake, Siderophores	Motility, Flagella	Secretion Systems	Toxins	Other (Unclassified)	Total Counts
A1	43	2	5	1	4	54	31	5	19	164
A2	45	2	4	1	1	84	58	2	21	218
A4	26	2	3	1	4	71	59	0	20	186
S2	46	2	2	1	1	85	59	2	22	220
S3	46	2	2	1	1	85	58	2	22	219
LMG24783	46	2	2	1	1	84	59	2	20	217
A-9	43	2	2	1	4	53	17	6	23	151

**Table 5:** Secretion system CDS counts per genome.

Genome	T2SS	T3SS	T4SS	T6SS
A1	13	0	1	17
A2	13	27	1	17
A4	14	35	1	9
S2	13	27	1	18
S3	13	27	1	17
LMG24783	13	27	1	18
A-9	14	0	1	2

## Data Availability

The original contributions presented in the study are included in the article/Supplementary Materials. Data supporting the findings of this study are available upon request from the corresponding author. Further inquiries can be directed to the corresponding author.

## Supplementary Materials

The following supporting information can be downloaded at: https://www.mdpi.com/article/10.3390/pathogens15040402/s1, Table S1: Sequences of primers and probe sets used; Table S2: Bacterial counts related to inoculum used for in vivo testing; Table S3: Predicted gene function for VF detected in *A. piscicola* A2 genome; Table S4: Predicted gene function for the O-antigen cluster found in the *A. piscicola* A2 genome.

## References

[1] Beaz-Hidalgo R., Latif-Eugenin F., Figueras M.J. (2013). The improved PCR of the fstA (ferric siderophore receptor) gene differentiates the fish pathogen *Aeromonas salmonicida* from other Aeromonas species. Vet. Microbiol..

[2] Austin B., Austin D.A., Dalsgaard I., Gudmundsdottir B.K., Hoie S., Thornton J.M., Larsen J.L., O’hici B., Powell R. (1998). Characterization of atypical *Aeromonas salmonicida* by different methods. Syst. Appl. Microbiol..

[3] Wiklund T., Dalsgaard I. (1998). Occurrence and significance of atypical *Aeromonas salmonicida* in non-salmonid and salmonid fish species: A review. Dis. Aquat. Org..

[4] Sun X.N., Wang Q., Wang Y.F., Tao Y., Zheng C.L., Wang M.H., Che M.Y., Cui Z.H., Li X.L., Zhang Q. (2023). Isolation and identification of vapA-absent *Aeromonas salmonicida* in diseased snakehead Channa argus in China. Int. Microbiol..

[5] Vazquez-Fernandez E., Chinchilla B., Rebollada-Merino A., Dominguez L., Rodriguez-Bertos A. (2023). An Outbreak of *Aeromonas salmonicida* in Juvenile Siberian Sturgeons (*Acipenser baerii*). Animals.

[6] Pavan M.E., Abbott S.L., Zorzopulos J., Janda J.M. (2000). *Aeromonas salmonicida* subsp. pectinolytica subsp. nov., a new pectinase-positive subspecies isolated from a heavily polluted river. Int. J. Syst. Evol. Microbiol..

[7] Beaz-Hidalgo R., Alperi A., Figueras M.J., Romalde J.L. (2009). *Aeromonas piscicola* sp. nov., isolated from diseased fish. Syst. Appl. Microbiol..

[8] Lagadec E., Mjolnerod E.B., Jensen O.M., Plarre H., Nylund A. (2024). Multiple Aeromonas strains isolated from Atlantic salmon (*Salmo salar*) displaying red skin disease signs in Scandinavian rivers. J. Fish Dis..

[9] Sernapesca (2025). Informe Con Información Sanitaria de Agua Dulce y Mar, Año 2024.

[10] Godoy M., Gherardelli V., Heisinger A., Fernandez J., Olmos P., Ovalle L., Ilardi P., Avendano-Herrera R. (2010). First description of atypical furunculosis in freshwater farmed Atlantic salmon, *Salmo salar* L., in Chile. J. Fish Dis..

[11] Godoy M., Montes De Oca M., Suarez R., Martinez A., Pontigo J.P., Caro D., Kusch K., Coca Y., Bohle H., Bayliss S. (2023). Genomics of Re-Emergent *Aeromonas salmonicida* in Atlantic Salmon Outbreaks. Microorganisms.

[12] Mancilla M., Ojeda A., Yuivar Y., Grandón M., Grothusen H., Oyarzún M., Bisquertt A., Ugalde J.A., Fuentes F., Ibarra P. (2025). Major antigenic differences in *Aeromonas salmonicida* isolates correlate with the emergence of a new strain causing furunculosis in Chilean salmon farms. Front. Cell. Infect. Microbiol..

[13] CLSI (2014). Methods for broth dilution susceptibility testing of bacteria isolated from aquatic animals. Approved Guideline.

[14] CLSI (2014). Performance Standards for Antimicrobial Susceptibility Testing of Bacteria Isolated from Aquatic Animals.

[15] Yu C.P., Chu K.H. (2011). Molecular quantification of virulence gene-containing Aeromonas in water samples collected from different drinking water treatment processes. Environ. Monit. Assess..

[16] Chen S. (2023). Ultrafast one-pass FASTQ data preprocessing, quality control, and deduplication using fastp. Imeta.

[17] Bankevich A., Nurk S., Antipov D., Gurevich A.A., Dvorkin M., Kulikov A.S., Lesin V.M., Nikolenko S.I., Pham S., Prjibelski A.D. (2012). SPAdes: A new genome assembly algorithm and its applications to single-cell sequencing. J. Comput. Biol..

[18] Schwengers O., Jelonek L., Dieckmann M.A., Beyvers S., Blom J., Goesmann A. (2021). Bakta: Rapid and standardized annotation of bacterial genomes via alignment-free sequence identification. Microb. Genom..

[19] Altschul S.F., Gish W., Miller W., Myers E.W., Lipman D.J. (1990). Basic local alignment search tool. J. Mol. Biol..

[20] Gilchrist C.L.M., Chooi Y.H. (2021). Clinker & clustermap.js: Automatic generation of gene cluster comparison figures. Bioinformatics.

[21] Yoon S.H., Ha S.M., Lim J., Kwon S., Chun J. (2017). A large-scale evaluation of algorithms to calculate average nucleotide identity. Antonie Van Leeuwenhoek.

[22] Meier-Kolthoff J.P., Goker M. (2019). TYGS is an automated high-throughput platform for state-of-the-art genome-based taxonomy. Nat. Commun..

[23] Liu B., Zheng D., Zhou S., Chen L., Yang J. (2022). VFDB 2022: A general classification scheme for bacterial virulence factors. Nucleic Acids Res..

[24] Ajmi N., Duman M., Ay H., Saticioglu I.B. (2025). Genomic and Pangenomic Insights into *Aeromonas salmonicida* subsp. oncorhynchi subsp. nov. Pathogens.

[25] Feldgarden M., Brover V., Gonzalez-Escalona N., Frye J.G., Haendiges J., Haft D.H., Hoffmann M., Pettengill J.B., Prasad A.B., Tillman G.E. (2021). AMRFinderPlus and the Reference Gene Catalog facilitate examination of the genomic links among antimicrobial resistance, stress response, and virulence. Sci. Rep..

[26] Alcock B.P., Huynh W., Chalil R., Smith K.W., Raphenya A.R., Wlodarski M.A., Edalatmand A., Petkau A., Syed S.A., Tsang K.K. (2023). CARD 2023: Expanded curation, support for machine learning, and resistome prediction at the Comprehensive Antibiotic Resistance Database. Nucleic Acids Res..

[27] Maiti B., Shetty M., Shekar M., Karunasagar I., Karunasagar I. (2011). Recombinant outer membrane protein A (OmpA) of *Edwardsiella tarda*, a potential vaccine candidate for fish, common carp. Microbiol. Res..

[28] Yi E.C., Hackett M. (2000). Rapid isolation method for lipopolysaccharide and lipid A from Gram-negative bacteria. Analyst.

[29] Hitchcock P.J., Brown T.M. (1983). Morphological heterogeneity among Salmonella lipopolysaccharide chemotypes in silver-stained polyacrylamide gels. J. Bacteriol..

[30] Ishiguro E.E., Ainsworth T., Trust T.J., Kay W.W. (1985). Congo red agar, a differential medium for *Aeromonas salmonicida*, detects the presence of the cell surface protein array involved in virulence. J. Bacteriol..

[31] Allard J.D., Bertrand K.P. (1993). Sequence of a class E tetracycline resistance gene from Escherichia coli and comparison of related tetracycline efflux proteins. J. Bacteriol..

[32] Vasquez I., Hossain A., Gnanagobal H., Valderrama K., Campbell B., Ness M., Charette S.J., Gamperl A.K., Cipriano R., Segovia C. (2022). Comparative Genomics of Typical and Atypical *Aeromonas salmonicida* Complete Genomes Revealed New Insights into Pathogenesis Evolution. Microorganisms.

